# Intoxication au plomb révélant une drépanocytose

**DOI:** 10.11604/pamj.2018.30.305.16222

**Published:** 2018-08-31

**Authors:** Ahmed Mougui, Imane El Bouchti

**Affiliations:** 1 Service de Rhumatologie, CHU Mohammed VI, BP 2360 principal, Avenue Ibn Sina, Marrakech, Maroc

**Keywords:** Drépanocytose, plomb, ostéonécrose aseptique, Sickle cell disease, lead, aseptic osteonecrosis

## Abstract

We here report the case of a 18 year old patient with two brothers who died from sickle cell anemia. He had a 5-day history of diffuse bone pain. He had drunk a preparation containing lead to obtain an analgesic effect without improvement. Radiographic examination showed a condensation due to the presence of lead in the digestive tract, associated with aseptic osteonecrosis of the two femoral heads on the X-ray of the pelvis (A) and of the vertebrae in H on the X-ray of the dorso-lumbar spine (B). These signs were suggestive of sickle cell anemia. The diagnosis was confirmed by hemoglobin electrophoresis test showing haemoglobin S at 79%.

## Image en médecine

Patient de 18 ans, ayant deux frères décédés par drépanocytose. Présente depuis 5 jours des douleurs osseuses diffuses. Il avait bu une préparation contenant du plomb dans un but antalgique, sans amélioration. Le bilan radiographique montre, une condensation liée à la présence du plomb dans le tube digestif, avec une ostéonécrose aseptique des deux têtes fémorales sur la radiographie du bassin face (A), et des vertèbres en H sur la radiographie du rachis dorsolombaire profil (B). Ces signes sont évocateurs de la drépanocytose. Le diagnostic est confirmé par l'électrophorèse d'hémoglobine montrant une hémoglobine S à 79%.

**Figure 1:**
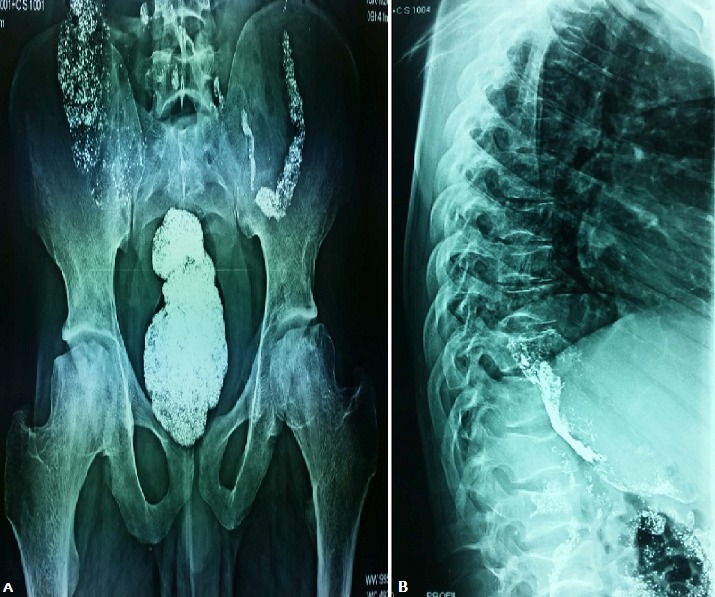
A) radiographie du bassin face montrant la condensation digestive et l'ostéonécrose aseptique des têtes fémorales; B) radiographie du rachis dorsolombaire montrant des vertèbres en H

